# Computational Screening of the Human TF-Glycome Provides a Structural Definition for the Specificity of Anti-Tumor Antibody JAA-F11

**DOI:** 10.1371/journal.pone.0054874

**Published:** 2013-01-24

**Authors:** Matthew B. Tessier, Oliver C. Grant, Jamie Heimburg-Molinaro, David Smith, Snehal Jadey, Andrew M. Gulick, John Glushka, Susan L. Deutscher, Kate Rittenhouse-Olson, Robert J. Woods

**Affiliations:** 1 Complex Carbohydrate Research Center and Department of Chemistry, University of Georgia, Athens, Georgia, United States of America; 2 School of Chemistry, National University of Ireland, Galway, University Road, Galway, Ireland; 3 Department of Biotechnical and Clinical Laboratory Sciences, State University of New York, Buffalo, New York, United States of America; 4 Department of Biochemistry, Emory University, Atlanta, Georgia, United States of America; 5 Hauptman-Woodward Institute, Department of Structural Biology, State University of New York, Buffalo, New York, United States of America; 6 Department of Biochemistry, University of Missouri, Columbia, Missouri, United States of America; Concordia University Wisconsin, United States of America

## Abstract

Recombinant antibodies are of profound clinical significance; yet, anti-carbohydrate antibodies are prone to undesirable cross-reactivity with structurally related-glycans. Here we introduce a new technology called Computational Carbohydrate Grafting (CCG), which enables a virtual library of glycans to be assessed for protein binding specificity, and employ it to define the scope and structural origin of the binding specificity of antibody JAA-F11 for glycans containing the Thomsen-Friedenreich (TF) human tumor antigen. A virtual library of the entire human glycome (GLibrary-3D) was constructed, from which 1,182 TF-containing human glycans were identified and assessed for their ability to fit into the antibody combining site. The glycans were categorized into putative binders, or non-binders, on the basis of steric clashes with the antibody surface. The analysis employed a structure of the immune complex, generated by docking the TF-disaccharide (Galβ1-3GalNAcα) into a crystal structure of the JAA-F11 antigen binding fragment, which was shown to be consistent with saturation transfer difference (STD) NMR data. The specificities predicted by CCG were fully consistent with data from experimental glycan array screening, and confirmed that the antibody is selective for the TF-antigen and certain extended core-2 type mucins. Additionally, the CCG analysis identified a limited number of related putative binding motifs, and provided a structural basis for interpreting the specificity. CCG can be utilized to facilitate clinical applications through the determination of the three-dimensional interaction of glycans with proteins, thus augmenting drug and vaccine development techniques that seek to optimize the specificity and affinity of neutralizing proteins, which target glycans associated with diseases including cancer and HIV.

## Introduction

Aberrant glycosylation is a hallmark of many diseases, including cancer[1], and can therefore provide a basis for disease diagnosis and staging, and may potentially be exploited for therapeutic intervention[2]. An established carbohydrate-based cancer marker is the Thomsen-Friedenreich (TF) antigen (Galβ1-3GalNAcα), which is typically found *O*-linked to serine or threonine residues. The TF antigen (also known as T antigen) has been associated with several human carcinomas, including those found in the pancreas, colon, and breast, and on this basis has been referred to as a pan-carcinoma marker[3], [4], [5], [6]. TF antigen is concealed from the immune system in normal adult tissues as a result of extension with larger glycan chains[5], [7]. In cancer, the cellular glycosylation machinery may be disrupted, leading to truncation of these chains and exposure of the TF antigen[8]. Here, we examine the specificity of a potentially diagnostic and therapeutic monoclonal antibody (mAb JAA-F11)[9] that was raised against the TF antigen[10]. JAA-F11 preferentially binds to tumor tissue over normal[11], and *in vivo* it enhances survival and decreases metastasis in the mouse 4T1 metastatic model[9], indicating a potential for this mAb to be used, after humanization, in cancer patient therapy.

The potential also exists for anti-carbohydrate mAbs, such as JAA-F11, to be used as diagnostic agents, however, the diversity of glycans present in eukaryotic organisms leads to the possibility for cross-reactivity among structurally similar carbohydrates, which may nevertheless have unrelated biological roles. Thus, it is particularly critical to determine the specificity of any reagent proposed for use in glycan-based disease-marker detection[12], [13], [14]_ENREF_11. Over the past decade, glycan microarray screening has gained wide-spread popularity as a technique for assessing carbohydrate-binding specificity. The largest glycan microarrays currently contain on the order of 600 members[15], enabling rapid assessment of binding specificity, and requiring far less protein and carbohydrate for the analysis than would be necessary for more detailed affinity measurements. Despite these advances the human glycome is far more diverse than even the largest experimental glycan array[13]. Thus, experimental screening of the entire human glycome is not yet feasible, leaving the potential for cross-reactive binding to go undetected. Moreover, although glycan array screening can provide specificity data for many glycans simultaneously, the data are difficult to relate directly to binding affinities, and do not provide insight into the structural mechanisms of a binding interaction.

Structural information is traditionally provided by experimental methods such as X-ray crystallography and NMR spectroscopy. Despite the importance of 3D structural data in defining structure-function relationships, neither NMR spectroscopy nor protein crystallography can be considered high throughput methods in this role. Additionally, both techniques face significant challenges when applied to the characterization of certain classes of protein-ligand complexes, such as those formed between antibodies and large glycans[16], [17]._ENREF_2 Issues such as glycan flexibility, structural heterogeneity, and challenges in the synthesis, isolation or crystallization of complex glycans contribute to difficulties in such studies. In addition, to enhance the likelihood of crystallization, and to facilitate NMR data interpretation, such studies typically employ only di- or tri-saccharide fragments, rather than the whole, intact glycan.

**Figure 1:**
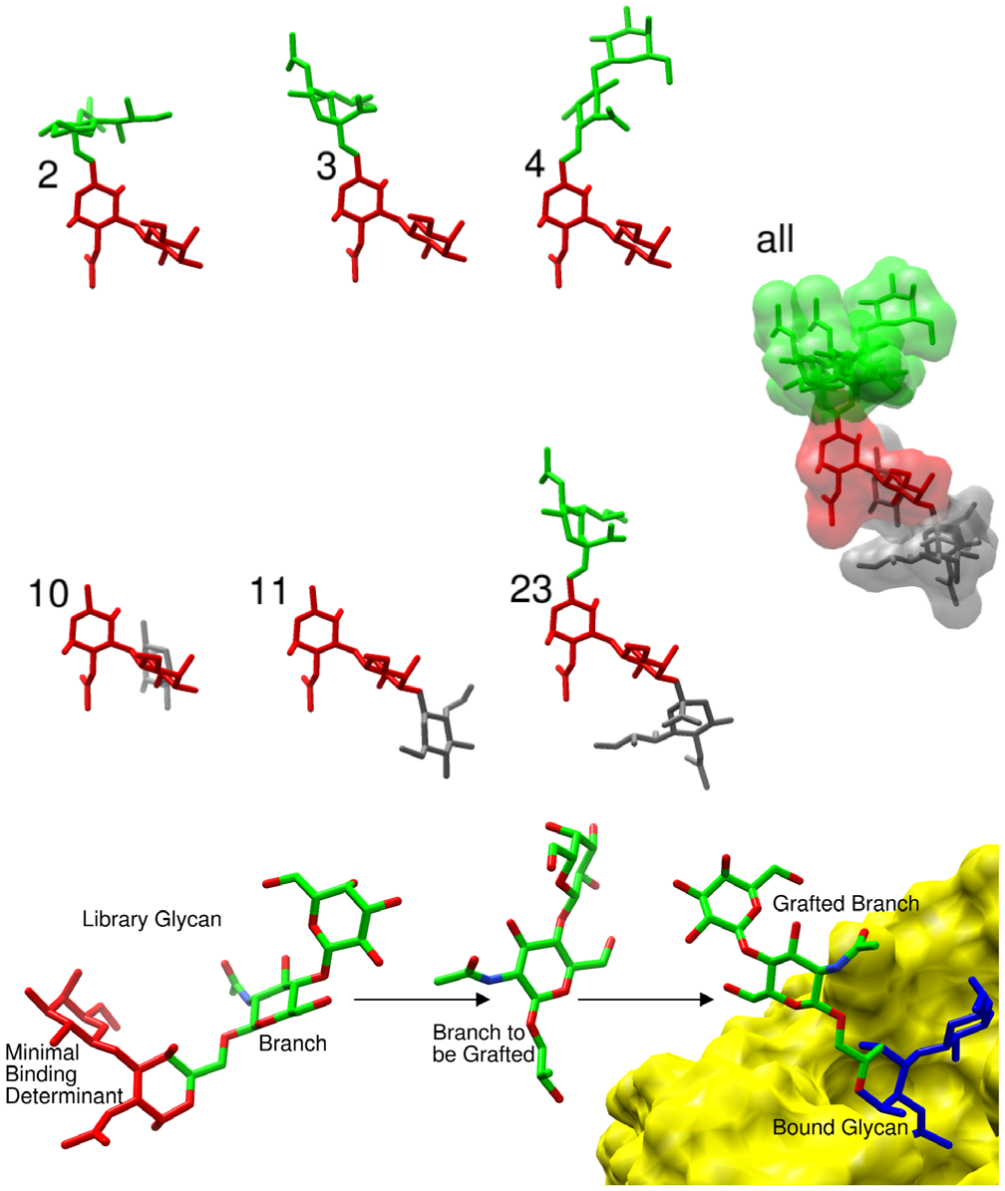
An illustration of Computational Carbohydrate Grafting (CCG) method applied to predict binding conformations of TF-containing glycans binding to the JAA-F11 antibody. **Upper**. Examples of glycans that bind to JAA-F11: Neu5Acβ2-6(Galβ1-3)GalNAcα (**2**); Neu5Acα2-6(Galβ1-3)GalNAcα (**3**); Galβ1-4GlcNAcβ1-6(Galβ1-3)GalNAcα (**4**), as well as non-binding sequences (**middle**): Fucα1-2Galβ1-3GalNAcα (10); GlcNAcβ1-3Galβ1-3GalNAcα (**11**); and Neu5Acα2-6(Neu5Acα2-3Galβ1-3)GalNAcα (**23**), showing the minimal binding determinant in red, the tolerated glycan branches in green, and the disallowed branches in grey. Also presented are the combined solvent-accessible surfaces from a superimposition of the sequences based on aligning the minimal determinant. **Lower**. In the grafting process branches from TF-containing glycans in the library are excised and spliced onto the bound minimal determinant. The grafted branches are then assessed for steric clashes with the antibody surface. This process is illustrated for the grafting of the glycan branch Galβ1-4GlcNAcβ1-6 (green carbon frame) from **4** onto the TF antigen in the JAA-F11 binding site (yellow solvent-accessible surface). Figures generated with Chimera [52].

Here we present a new technology, Computational Carbohydrate Grafting (CCG) that is complementary to glycan array screening, NMR spectroscopy and crystallography. CCG leverages available 3D structural data for carbohydrate-protein complexes, with virtual glycan library screening to generate 3D models of glycan-protein complexes. We use CCG to predict the binding specificity of JAA-F11, and demonstrate that the theoretical predictions are fully consistent with experimental specificity data for the same antibody generated by screening against an experimental glycan array. In contrast to traditional virtual screening, which would attempt to dock the entire glycan into the binding site, CCG splices the glycan branches onto the appropriate positions in a fragment (TF antigen in this case, **Fig. 1**) of the glycan present in a protein-carbohydrate complex. The 3D orientations of the grafted branches relative to the fragment (or minimal binding determinant) are generated on the basis of established carbohydrate conformational preferences[18]. Quantification of any steric overlaps between the grafted glycan and the protein surface enables discrimination between potential binding partners and non-binders. It should be noted that the CCG analysis does not rank the ligands in terms of theoretical affinities. Nevertheless, the power of the CCG method is the rapid identification of a subset of putative binders, which can subsequently be examined in more detail either experimentally or theoretically. By grafting the virtual glycan structures onto a bound carbohydrate motif, a level of speed and accuracy in the prediction of the 3D structures is achieved that would otherwise be impossible using either traditional virtual screening or experimental techniques alone. In addition, CCG facilitates the screening of vast libraries of glycans that can encompass the entire known human glycome, as well as synthetic or hypothetical structures, extending the CCG screening capability far beyond the scope of current experimental glycan microarrays.

A CCG analysis of JAA-F11 was performed against a virtual array of 1,182 TF-containing human glycans, extracted from a library of glycan 3D structures (GLibrary-3D), comprising the known human glycome as present in the GlycomeDB database (www.glycome-db.org)[19]. The virtual screening employed a crystal structure of the Fab (**Table 1**); however, co-crystals of the Fab–TF antigen complex proved to be elusive, thus computational docking of the minimal motif (TF disaccharide) was performed. Experimental support for the predicted orientation of the TF disaccharide in the binding site was provided by data from saturation-transfer difference (STD) NMR experiments on the mAb-TF complex (see **Results S1**, **Figures S1 and S2**). The docked orientation of the minimal determinant was consistent with the STD NMR data, and subsequent CCG analysis employing this complex fully explained the observed binding specificity of this mAb from experimental glycan array screening. In addition, the CCG screening led to the prediction of a small number of putative binders that are not present on the current experimental arrays. Taken together, the CCG analysis and experimental glycan array data confirm the specificity of JAA-F11 for TF antigen and certain extended core-2 type mucins.

**Table 1:** Data collection and refinement statistics for Fab JAA-F11.

Data collection	
Space group	P4_3_2_1_2
Cell dimensions	
*a*, *b*, *c* (Å)	94.2, 94.2, 95.0
α, β, γ (°)	90°, 90°, 90°
Resolution (Å)	30.0–2.1
*R*_merge_ (%)	9.6 (43.3)^a^
<I/σ(I)>	9.6 (2.0)
Completeness (%)	99.0 (98.2)
Redundancy	10.5 (9.5)
Refinement	
Resolution (Å)	30.0–2.1
Number of reflections	24063
*R*_work_/*R*_free_	18.5/26.2 (19.9/29.9)
Number of atoms	
Protein	3342
Ligands	20
Water	176
Average *B*-factors	
Protein (Å^2^)	21.0
Ligands (Å^2^)	54.8
Water (Å^2^)	24.3
RMS deviations	
Bond lengths (Å)	0.02
Bond angles (°)	1.9

a Values in parentheses are for highest-resolution shell (2.2–2.1 Å).

## Results

### Crystal Structure of Fab JAA-F11

The unliganded crystal structure of Fab JAA-F11 was determined at 2.1 Å (see **Table 1**), and consists of Leu^L1^-Asn^L217^ of the variable light chain (V_L_) and Ala^H1^-Arg^H218^ of the variable heavy chain (V_H_), as well as 176 water molecules.

**Figure 2:**
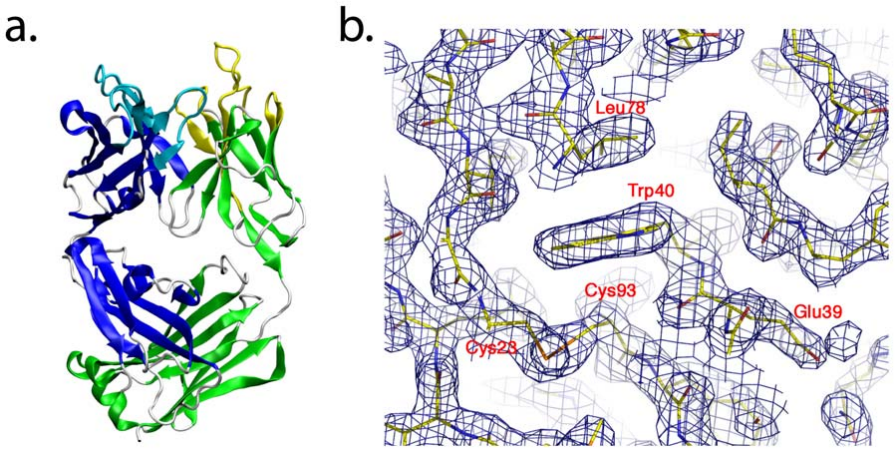
Illustrations of the 3D structure of the JAA-F11 antibody as determined by X-ray crystallography. **a**. The overall JAA-F11 Fab fold in ribbons (heavy chain residues in blue, light chain in green) indicating the CDRs (heavy chain in light blue, light chain in yellow). **b**. Representative electron density of the final map for a region of the light chain; the map is calculated with coefficients of the form 2Fo-Fc, and contoured at 1.2. The conserved disulfide bond between Cys^L23^ and Cys^L93^ is shown.

A ribbon diagram of the Fab shows that it displays the typical overall fold of a Fab fragment (**Fig. 2A**). The quality of the electron density for a region of the light chain is also provided (**Fig. 2B**). Complementarity determining regions (CDRs) in the Fab were assigned to canonical structure class 1 for loops L2 (Lys^L55^ to Ser^L61^), L3 (Phe^L94^ to Thr^L102^), and H1 (Thr^H31^ to His^H35^), while loops L1 (Arg^L24^ to Glu^L39^) and H2 (Phe^H50^ to Asp^H65^) belong to canonical structures 4 and 2, respectively. CDR H3 consisted of residues Ser^H99^ to Phe^H107^ and could not be assigned a canonical conformation. Together these CDRs form a canyon shaped[20] binding pocket of 712 Å^3^ volume.

### 3D Model of the Minimal Determinant–Fab complex

Computational docking of the TF disaccharide was performed with AutoDock 3.05[21] to identify possible poses of the antigen in the Fab JAA-F11 binding site. The crystal structure of the Fab and a 3D structure for the TF antigen, obtained from GLYCAM-Web (www.glycam.org)[22], were employed in the docking. During docking, the φ and ψ torsion angles of the glycosidic linkages were maintained in the low energy conformation generated by energy minimization with the GLYCAM06 force field[23], while all hydroxyl and C_5_-C_6_ bonds were allowed to rotate freely. Fifty poses were obtained (**Table S1**) and ranked in terms of predicted interaction energy and pose clustering. Clustering of the docking results was performed, based on placement and orientation of each pose relative to the protein.

**Figure 3:**
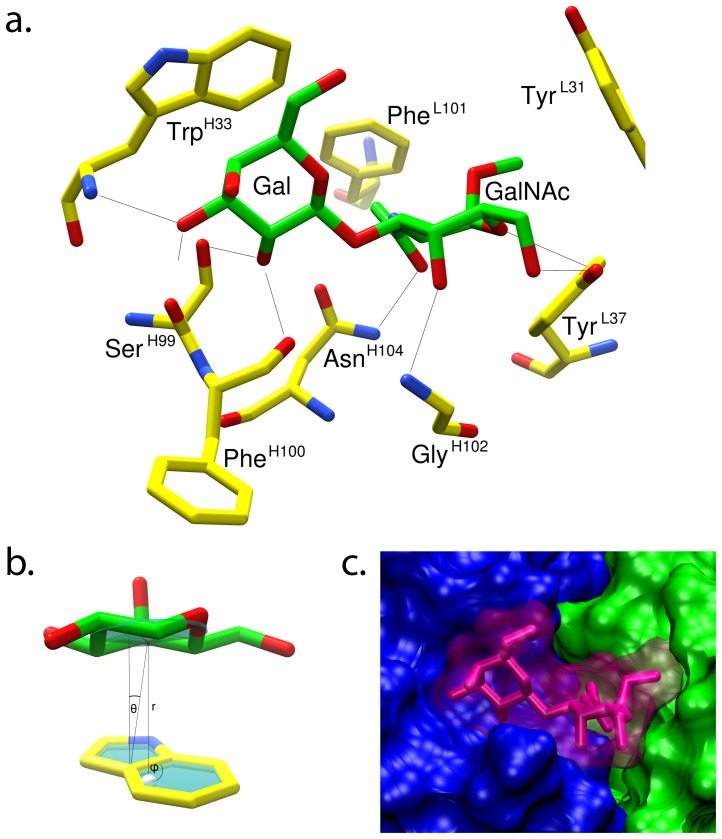
The binding interactions predicted from the docked model of the TF-disaccharide bound to JAA-F11. **a.** Validated model of the bound minimal determinant (green carbon frame) in the mAb binding site, hydrogen atoms removed for clarity. Protein residues (yellow carbon frame) involved in hydrogen bonds (black lines) or hydrophobic interactions with the TF antigen are shown. **b.** Depiction of the stacking interaction between the Gal and the Trp^H33^ (r  =  4.0 Å, θ  =  12.1°, φ  =  90.3°); the geometry of this interaction is comparable to literature values[53] (lower left). **c.** Solvent accessible surface of the bound minimal determinant (magenta) and the Fab (showing V_H_ and V_L_ regions in blue and green, respectively.

**Figure 4:**
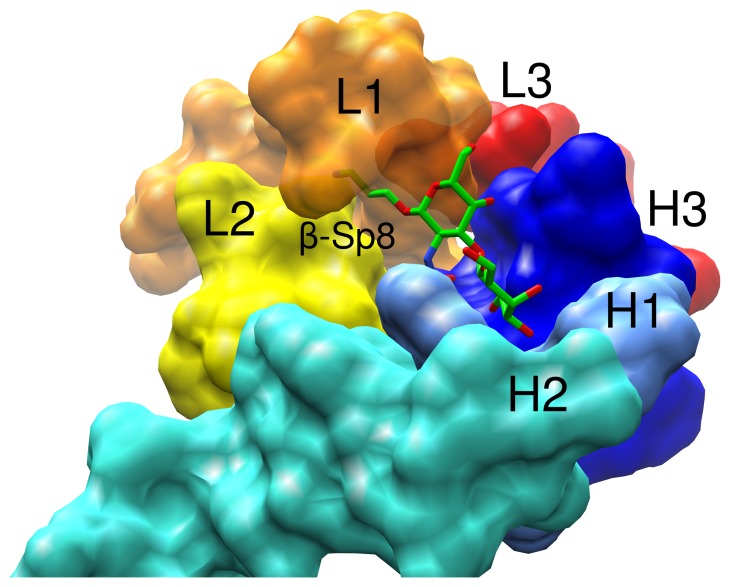
Solvent accessible surface of the CDRs illustrating the predicted overlap between a β-linked aglycon in the TF disaccharide and CDR L1, responsible for ensuring the α-TF specificity of JAA-F11.

The most highly populated cluster (**see Table S1**) also exhibited the lowest average energy of all clusters and contained the lowest energy pose (pose 1, **Fig. 3**). In this optimal pose, four hydrogen bonds were formed between the protein and oxygen atoms in the terminal Gal residue; Ser^H99^ O_γ_ to O-2, Phe^H100^ O to O-2, Trp^H33^ N to O-3 and Ser^H99^ O to O-3. Four additional hydrogen bonds were formed with the GalNAc residue; Asn^H104^ N_δ_ to the carbonyl oxygen atom of the *N*-acetyl group, Gly^H102^ N to O-4, and Tyr^L37^ OH to O-5 and O-6 (**Fig. 3A**). These data are consistent with earlier conclusions that neither of the primary hydroxyl groups was involved in binding[10]. The presence of an aromatic stacking interaction between the Gal residue and Trp^H33^ was also observed, which is a common feature in carbohydrate-protein complexes (**Fig. 3B**)[24]. Based on this structure for the immune complex, the important observation that this mAb binds α-linked TF antigen, but not β-TF, yielding tumor specificity, could be rationalized on the basis that a β-linkage at the reducing terminus would result in overlaps between the aglycon and several residues in CDR L1, including, Tyr^L31^, Ser^L32^, and Asn^L33^ (**Fig. 4**).

### Antigen pose validation through STD-NMR

**Figure 5:**
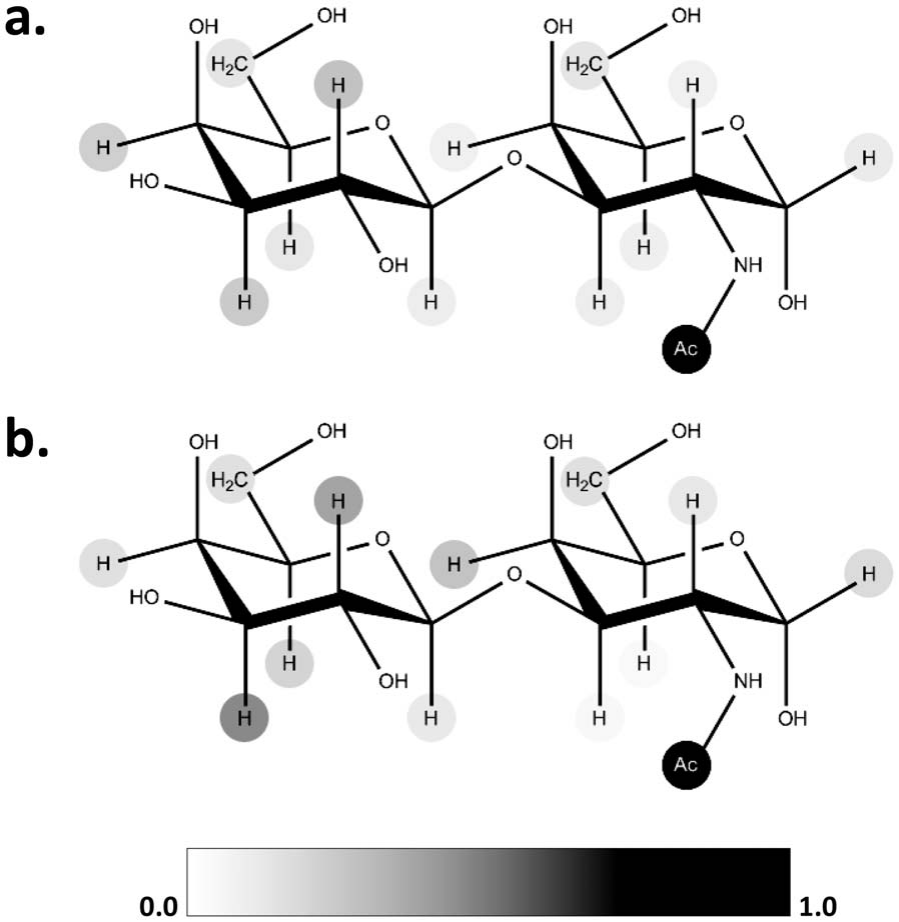
Graphical representation of the normalized NMR-STD data for TF antigen in complex with JAA-F11, experimental (a) and theoretical (b).

Pose 1 from the docking was both top ranked in terms of cluster population, and lowest energy, and explained the specificity of this mAb for α-linked TF antigen. To further support the theoretical docked model, experimental confirmation was sought from saturation transfer difference (STD) NMR experiments. As the name implies, STD experiments detect the difference in nuclear Overhauser enhancement (nOe) magnetization transfer from the irradiated protein to the bound and free states of a ligand. The relative enhancements of the proton signals in the bound state of the ligand are proportional to the proximity of those protons to protons in the protein. Thus, STD data provide important insight into the bound orientation of the ligand. This information permits a direct comparison between the experimental STD enhancements, and those derived from the theoretical orientation produced by docking the ligand in the Fab–TF antigen complex. STD enhancements were computed from the theoretical complex following an adaptation of the isolated spin-pair approximation (ISPA)[25], frequently employed for estimating nOe values. In ISPA, the assumption is made that the nOe intensities are proportional to *R_ij_^−6^*, where *R_ij_* is the inter-proton distance between spins *i* and *j*. Here, STD intensities were derived for each proton in the ligand based on the sum of the *R_ij_^−6^* values between each proton in the Fab fragment (*i*) and those in the ligand (*j*). In the case of methyl groups, contributions from each proton were computed in the sum of *R_ij_^−6^* values. The agreement between the predicted and experimental STDs is illustrated in **Figure 5**, which shows that the strongest interactions with the protein surface involve the *N*-Acetyl methyl group (GalNAc) followed by H-2, H-3 and H-4 (Gal). Both the STD data and the theoretical structure indicate a key role for the *N*-acetyl moiety in the binding of TF antigen to JAA-F11. Each of the other poses obtained by docking was subjected to a similar analysis, however, only pose 1 resulted in satisfactory agreement between the theoretical and experimental STD data (**Figure S1**). The theoretical model based on pose 1 was therefore adopted as a basis for the subsequent CCG specificity analysis.

### CCG specificity predictions and comparison to experimental specificity data

The number of glycan sequences included in the CCG specificity analysis is not limited by physical constraints, and the method can therefore be extended far beyond the capacity of current experimental arrays. Even the largest glycan arrays to date likely represent only 10–15 percent of the known human glycome[13], although even with a relatively small coverage it is possible to include a representative diversity of glycans[15]. The present virtual library of 3D glycan structures (GLibrary-3D) contains over 7,000 glycans, represented by more than 200,000 unique 3D rotamers, spanning the known human glycome.

It is important to note that because of the specificity of glycosyltransferases, not all permutations of glycosidic linkages are biologically relevant in all contexts. For example, the Neu5Acα(2-6)Gal linkage is not possible when the Gal residue is present in mucins containing the core-1 (TF) disaccharide, but is commonplace in complex *N*-linked glycans. Biological relevance was assessed after CCG analysis for the glycans predicted to be binders. Any non-natural sequences were discarded from further consideration.

To ensure that CCG identified only true non-binders (*i.e.* glycans whose lack of binding arose solely from steric overlaps with the mAb), the analysis was performed without explicit consideration of the spacer type used on the experimental array[26]. In the case of glycans containing flexible linkages, each stable rotamer was generated and analyzed for steric clashes with the protein surface. Overlaps were quantified in terms of the area of the van der Waals overlap between the atoms in the glycan and the protein. In all cases, methyl glycosides were employed and confirmed the exclusive tolerance for the α-configuration at the reducing terminus of the TF antigen; all β-anomers led to steric overlaps between the methyl and the mAb surface. The virtual screening indicated that in addition to the TF antigen, the only other glycans that did not make significant steric clashes with the mAb surface were those that contained branches emanating from the O-6 positions of either the TF Gal or GalNAc residues (**Table 2**).

**Table 2:** Human glycans predicted to fit^a^ into the binding site of JAA-F11, in addition to those present on the CFG v4.0 array.

GlycomeDB ID	Glycan Sequence
10743, 18135, 3618, 32649, 32532	Galβ1-4GlcNAcβ1(-3Galβ1-4GlcNAcβ1)_1-4_-6**(Galβ1-3)GalNAc**
32608	Neu5Acα2(-3Galβ1-4(Fucα1-3)GlcNAcβ1)_3_-6**(Galβ1-3)GalNAc**
10752, 22152	Galβ1-4(Fucα1-3)GlcNAcβ1-6**(Galβ1-3)GalNAc**
3184, 10753	Fucα1-2Galβ1-4(Fucα1-3)GlcNAcβ1-6**(Galβ1-3)GalNAc**
985	Galβ1-3GlcNAcβ1-3Galβ1-4GlcNAcβ1-6**(Galβ1-3)GalNAc**
1271, 13480, 10751, 21997	Fucα1-2Galβ1-4GlcNAcβ1-6**(Galβ1-3)GalNAc**

a CCG analysis indicated no clashes between these glycans and the JAA-F11 surface.

In terms of the application of JAA-F11 as a therapeutic or diagnostic reagent, despite the large number of human glycans present in GLibrary-3D, only core 2 mucins with sialic acid, or polylactosamine extensions, at the 6-position in the core GlcNAc residue were predicted to bind, in addition to those present in the glycan array (**Table 3**). Thus, CCG screening of the virtual library predicted that JAA-F11 would be specific for TF antigen and a very limited subset of TF-containing human glycans.

**Table 3:** Comparison of theoretical and experimental specificity data for mAb JAA-F11 with glycans containing the TF motif present in the CFG v4.0 glycan array.

ID	Glycan Sequence	Theoretical	Experimental RFU^a^		
		Clash Score^b^	Sp8^c^	Sp14	Sp0
**1**	**Galβ1-3GalNAcα**	0	98	0	---^d^
**2**	Neu5Acβ2-6**(Galβ1-3)GalNAcα**	0	81	---	---
**3**	Neu5Acα2-6**(Galβ1-3)GalNAcα**	0.1	78	0	---
**4**	Galβ1-4GlcNAcβ1-6**(Galβ1-3)GalNAcα**	0	52	0	---
**5**	GlcNAcβ1-6**(Galβ1-3)GalNAcα**	0	51	0	---
**6**	Neu5Acα2-3Galβ1-4(Fucα1-3)GlcNAcβ1-6**(Galβ1-3)GalNAcα**	0	---	0	---
**7**	Neu5Acα2-3Galβ1-4GlcNAcβ1-6**(Galβ1-3)GalNAcα**	0	---	0	---
**8**	(3S)**Galβ1-3GalNAcα**	2.8	0	---	---
**9**	Fucα1-2**Galβ1-3GalNAcα**	7.6	0	0	---
**10**	GlcNAcβ1-2**Galβ1-3GalNAcα**	11.6	0	---	---
**11**	GlcNAcβ1-3**Galβ1-3GalNAcα**	12.4	0	---	---
**12**	6S(Neu5Acα2-3**Galβ1-3)GalNAcα**	17.3	0	---	---
**13**	Neu5Acα2-3**Galβ1-3GalNAcα**	17.4	0	0	---
**14**	Neu5Acα2-6(Neu5Acα2-3**Galβ1-3)GalNAcα**	17.6	0	0	---
**15**	KDNα2-3**Galβ1-3GalNAcα**	13.8	---	0	---
**16**	Neu5Acα2-3Galβ1-4GlcNAcβ1-6(Neu5Acα2-3**Galβ1-3)GalNAcα**	17.4	---	0	---
**17^e^**	Neu5Acα2-3Galβ1-4(Fucα1-3)GlcNAcβ1-6(Neu5Acα2-3**Galβ1-3)GalNAc**	17.5	---	0	---
**18^e^**	GlcNAcα1-4**Galβ1-3GalNAc**	6.1	---	0	---
**19^e^**	**Galβ1-3GalNAcα**1-3(Fucα1-2)Galβ1-4GlcNAc	5.1	---	---	0
**20^e^**	**Galβ1-3GalNAcα**1-3(Fucα1-2)Galβ1-4Glc	5.3	---	---	0
**21**	Fucα1-2**Galβ1-3GalNAcα**1-3(Fucα1-2)Galβ1-4GlcNAcβ	12.6	---	---	0
**22**	Fucα1-2**Galβ1-3GalNAcα**1-3(Fucα1-2)Galβ1-4Glcβ	12.8	---	---	0
**23**	GalNAcα1-3(Fucα1-2)**Galβ1-3GalNAcα**1-3(Fucα1-2)Galβ1-4GlcNAcβ	26.3	---	---	0

a Normalized RFUs averaged over all protein concentrations (0.1, 5 and 200 μg/mL) and over multiple values for the same glycan, when present on the CFG array, see Methods. ^b^Relative van der Waals overlap, see Methods. ^c^Spacers, Sp0: –(CH_2_)_2_NH_2_; Sp8: –(CH_2_)_3_NH_2_; Sp14: -threonine. ^d^Not present on glycan array. ^e^Reducing anomeric configuration undefined on the CFG array, α-configuration assumed for the CCG analysis.

To provide experimental confirmation of the specificity of JAA-F11, the mAb was screened against the Consortium for Functional Glycomics (CFG) printed glycan array (v4.0) at three protein concentrations (0.1, 5.0 and 200 μg/mL, **Table 3**, and **Table S2**). As expected, the mAb displayed selectivity for the TF disaccharide at all concentrations, however, it also reacted with four other glycans that contain the TF disaccharide in their sequence. At the lowest antibody concentration only three strong binders were observed. Only one non-TF glycan (Galα1-3GalNAcα) bound well, but only at the highest concentration of the antibody, indicating that the interaction is likely to be non-specific[14]. Thus, while the TF disaccharide is the minimal binding and preferred determinant for this mAb, it is not the exclusive ligand.

It is important to note that little is known about the cell-surface densities, abundances, or tissue distributions, for any of the putative binders other than the TF disaccharide. And only in the case of the TF disaccharide has the alteration of these properties in disease states, such as cancer, been examined. Thus, not all binders are necessarily biologically significant for this mAb. This fact may explain why JAA-F11 has a demonstrated specificity for tumor tissue over normal in mice[11].

### Effect of Glycan Spacers

The nature of the chemical conjugation of glycans to an array surface can lead to false negative binding. This is presumably because the spacer moiety alters either the presentation or accessibility of the glycan, or is itself in overlap with the protein surface[27]. By including multiple replicates of a glycan in the array, each conjugated through different linkers, it is possible to readily identify such spacer effects. Indeed, the data in **Table 3** demonstrate that conjugation of any of the high affinity binders via linker Sp14 completely abrogates binding (glycans **1**, **3–5**). Thus, it was not possible to determine on the basis of the experimental array data alone, whether a glycan that was conjugated solely via Sp14 (**6**, **7**, **15–18**) might in fact be a binder if it were conjugated through a non-interfering linker. Similarly, none of the glycans linked through Sp0 (**19–23**) bound to the mAb. Unlike the case of Sp14, there is no evidence from the experimental data to confirm whether or not Sp0 is itself interfering with binding. However, given the similarity of spacers Sp8 (–(CH_2_)_3_NH_2_) and Sp0, (–(CH_2_)_2_NH_2_), it is reasonable to infer that Sp0 would likely be non-interfering.

The CCG analysis provides a complementary structure-based approach for identifying linkage effects, correctly predicting each of the five glycans (**1**–**5**) confirmed to bind by glycan array screening, as well as all of the true non-binders (**8**–**23**). Glycans **6** and **7** (Sp14) were predicted to be binding partners for JAA-F11, which may not be unexpected, given their structural similarity to other binding glycans (**4** and **5**). What is notable in the case of **6** and **7**, however, is that the theoretical analysis independently identified a potential linker effect for Sp14. The present analysis clearly indicates a role for CCG in identifying putative false negative binding, and additionally draws attention to the need for printing experimental arrays with as many glycan–linker permutations as possible.

## Discussion

Carbohydrate-specific antibodies have a key role as diagnostic and therapeutic agents[28], [29], yet these interactions are some of the most challenging to characterize using traditional structural biology methods. In addition, glycan chemical sythesis remains a laborious undertaking. The CCG method requires minimal experimental 3D structural data, and can be used to both guide the prioritization of chemical synthethic efforts and to provide important insight into the structural basis for biological recognition and specificity. Once the 3D structure of the bound minimal determinant is established, through either theoretical or experimental methods, or a combination of both as employed here, CCG can be used to screen an effectively unlimited range of glycans that contain this minimal determinant. Experimental microarray data can subsequently be employed to provide cross-validation for a sub-set of the CCG predictions. While crystallographic analyses of such complexes may remain challenging, the predicted structures can potentially be corroborated using a number of experimental approaches, including, STD NMR, site-directed mutagenesis, as well as additional binding assays for predicted ligands or ligand analogs.

Experimentally-consistent structures for Fab–glycan complexes not only provide a basis for defining and predicting specificity, they also facilitate structure-based strategies for the directed evolution of antibodies with either varied specificity or affinity. In the case of JAA-F11, the 3D model can be further employed to guide the rational design of peptide mimotopes of the carbohydrate, for use as immunogens[30], [31], [32], [33], as well as to guide the selection of key residues to be included during antibody humanization. Humanization of a mouse mAb by insertion of the CDRs into a human antibody scaffold, can lead to changes in the 3D conformation, particularly in the interface between the variable light and heavy chains[34], with a corresponding loss of affinity or specificity. As the framework regions that support and give structure to the binding site are altered during the humanization process, the resulting Ab may have a reduced affinity or specificity for the target due to conformational changes in the binding site. CCG provides an opportunity to predict the effects that structural changes or point mutations have on antigen specificity and provides a structural basis from which to assess those CDRs and antigen contacts that must be maintained during antibody humanization (**Fig. 3**).

Several caveats to the applicability of the CCG method require consideration. The first relates to the requirement for a 3D structure of the minimal glycan determinant in the binding site. Inaccuracies in this complex will degrade the reliability of the theoretical specificity predictions. In the case of JAA-F11, automated docking was able to generate a 3D model for the immune complex that was consistent with data from STD-NMR experiments performed on the same system. In addition, specificity data from experimental glycan array screening may be employed as filters to eliminate experimentally-inconsistent poses predicted by automated docking (**Table S3**). This is a novel and enabling combination of two complementary high-throughput methods. Secondly, the scope and utility of the virtual glycan library is directly dependent on the content of experimental glycomic databases. The predictive power of virtual screening will improve as the number of experimentally confirmed glycans and their detailed characterization and annotation increases. Thirdly, CCG is a high-throughput screening method that currently treats the ligand and receptor as rigid, and thus ignores the potential for induced fit to enable binding. Fourthly, the method predicts only the potential for glycans to bind to a particular receptor; it does not take into account the relative affinity of the interaction, and, just as in the case of experimental array screening, neither the natural abundance nor the cellular localization of the glycans are considered. The determination of these properties will be essential for placing binding specificity data into biologically relevant contexts. Finally, unlike experimental glycan array screening, the CCG approach requires that the minimal binding determinant be known.

Over the past decade, data from glycan array screening initiatives, such as from the CFG, have confirmed the importance of glycans and glycan binding proteins in infectious diseases, cancer metastasis and progression, immune cell interaction, and congenital diseases, such as muscular dystrophy. Concurrently, glycomics studies employing mass spectrometry have greatly increased our knowledge of the diversity of the human glycome and its alteration in disease states. CCG provides a new tool to aid in the efficient translation of this information into the practical development of therapeutics and diagnostics.

## Materials and Methods

### Crystallization of JAA-F11 Fab and Structure Determination

The JAA-F11 IgG_3_ antibody was purified by triple ammonium sulfate precipitation, followed by Cibacron Blue 3GA agarose gel and hydroxyapatite column chromatography. Digestion and purification of the Fab utilized papain digestion followed by separation on a Protein A column. Purified JAA-F11 Fab fragment[10] was subjected to crystallization screening using the high-throughput crystallization screening facility at the Hauptman-Woodward Institute[35]. Multiple conditions were tested for optimization with hanging drop vapor diffusion. Final crystals were grown by vapor diffusion using a precipitant containing 26% PEG 5000, 50 mM lithium bromide, and 50 mM sodium citrate (pH 4.0). Crystals appeared in 3-4 days. Crystals were cryoprotected by transferring the crystal to solutions of mother liquor containing incrementally higher solutions of ethylene glycol (8, 16, and 24%) and flash cooled in liquid nitrogen. Data were collected at the A1 beamline of the Cornell High Energy Synchrotron Source and scaled with HKL2000[36]; the intensities were converted to structure factors using the TRUNCATE program of the CCP4 suite[37]. The structure was solved by molecular replacement with MOLREP[38]. Multiple Fab antibody fragments were tested as search models. A satisfactory solution was found using a search model consisting of the heavy and light chains from 1CLZ, the crystal structure of an antitumor directed antibody that recognizes the Lewis Y tetrasaccharide[39]. The molecular replacement solution was refined through iterative manual model building with COOT[40] and maximum likelihood refinement with REFMAC5[41]_ENREF_39. Diffraction and refinement statistics are presented in **Table 1**. The binding pocket volume was determined with the fpocket software package[42]. The final structure factors and coordinates are deposited with the Protein Database (PDB ID 3GNM).

### Glycan Array Screening

A sample of antibody JAA-F11, isolated by ammonium sulfate precipitation, was submitted to the Consortium for Functional Glycomics (CFG) for screening at concentrations of 0.1, 5.0, 5.0 and 200 µg/mL on version 4.0 of the glycan array. Fluorescence was obtained by detecting the antibody with an Alexa Fluor-488 labeled anti-mouse IgG (Invitrogen) at 5.0 µg/ml. Data collection and interpretation methods have been reported[15] and are available on the CFG website (www.functionalglycomics.org).

### Saturation Transfer Difference NMR

A sample of JAA-F11 antibody (6.7 µM, two binding sites per dimer) and TF disaccharide from Carbosynth, (1.9 mM, approximately equal amounts of α and β anomers at the reducing terminus) was prepared in buffered D_2_O, giving an approximate molar ratio of 100**∶**1 for each anomer per binding site. The JAA-F11 antibody was obtained from the Rittenhouse-Olson lab and the free TF-disaccharide was purchased from Carbosynth. STD data were collected on an 800 MHz Varian (Agilent) Inova spectrometer at 25 °C using the double pulsed field gradient spin echo[43] method for water suppression. Antibody protons were selectively irradiated using a train of 50 ms Gaussian pulses at 0.5 ppm and a difference spectra produced by subtracting a reference spectrum irradiated at 25 ppm[44]. A total of 2048 scans were acquired for each interleaved spectrum. Different irradiation times were obtained at 0.5, 1.0, 1.5, 2.0, 3.0, 4.0, and 8.0 seconds to obtain a build-up curve (**Figure S2**). Data were processed and integrated using Mnova software[45]. The 1.0 s irradiation time was used to compare to the docking model results.

### Virtual Docking

Docking was performed with AutoDock 3.05[21]. Waters of crystallization were removed from the Fab structure of JAA-F11 before assigning partial charges to the protein (Kollman)[46] and to the TF disaccharide (Gasteiger)[47]. Initial 3D models for the TF disaccharide were generated using the carbohydrate 3D structure generation tools at GLYCAM-Web (www.glycam.org)[22] which energy minimizes the glycan in implicit solvent using the GLYCAM06 force field[23]. The final structure can be characterized by the values for the glycosidic linkage: φ (H_1_-C_1_-O_3_-C_3_,)  =  61.5° and ψ (C_1_-O_3_-C_3_-H_3_)  =  6.2°, which are consistent with the dominant solution NMR conformation[48]. Torsion rotation within the epitope was limited to the exocyclic free rotors (H-O and C5-C6 bonds); all other torsions were frozen at the values of the initial 3D model. The docking region was defined so as to include all the hypervariable loops, by centering a grid box (33 Å per side) on the sidechain nitrogen of Asp^H100^. A cubic grid spacing of 0.375 Å was employed. Docking was performed using the Lamarckian Genetic Algorithm[49] with a population size of 150 and 2.5 million energy evaluations in each of 50 docking runs and a clustering cutoff of 2.0 Å was employed.

### Creation of GLibrary-3D

Glycans to be included in the virtual glycan library (GLibrary-3D) were each selected from GlycomeDB (www.glycome-db.org)[19], which is an online database that currently contains 3,570 *N*- and *O*-linked glycan sequences found in humans, of which 3,086 contained sufficient information to be converted into 3D structures. Because most of the structures contained in GlycomeDB were determined using mass spectrometric techniques, not all reported sequences included sufficient information to uniquely define the glycan. For example, many sequences do not include information regarding inter-residue linkage positions, and these were generally excluded from the virtual library. However, in the case of certain human glycan sequences, which display only a limited number of linkage possibilities, such as the disaccharides Neu5Acα(2-3/6)Gal or Galβ(1-3/4)GlcNAc, each linkage permutation was constructed. Additionally, on the basis of known glycan structures, all ring types were assumed to be pyranose. These assumptions resulted in a total of 7,127 unique putative human glycan sequences. For glycans containing 1-6 or 2-6 linkages, each stable rotamer of the ω-angle (+/-60°, 180°) was generated. Additional rotamers were built for the φ-angles in 2-3 linkages (-60° and 180°)[50], leading to a library of 207,693 glycan 3D structures (GLibrary-3D). Glycan sequences were converted to 3D structures using an automated version of the Carbohydrate Model Building Tool of GLYCAM-Web (www.glycam.org)[22].

### Computational Carbohydrate Grafting (CCG)

All putative human glycans containing the TF disaccharide (1,182 glycans) were extracted from GLibrary-3D. The glycan branches to be grafted onto the minimal determinant were then translated and rotated as required in order to ensure correct relative alignment of the branch with respect to the minimal determinant, as defined on the basis of glycosidic bond lengths and angles (**Fig. 1**). The φ- and ψ-glycosidic torsion angles of the newly-formed linkage, were assigned on the basis of known carbohydrate conformational properties[18]. In the case of glycans that contained torsion angles known to populate more than one stable rotamer, such as 1-6 or 2-6 linkages, each rotamer was generated and treated as an independent molecule in the grafting process leading to a total of 3,109 rotamers. Once assembled, the intact glycan was energy minimized with the GLYCAM force field as described above.

Steric overlaps between the grafted branches and the protein surface were determined from the area of overlap of the atomic van der Waals surfaces[51]. As even small van der Waals overlaps can lead to very high repulsion energies, in a rigid molecular alignment, clash scores were not determined energetically. To assess the significance of the clash, the van der Waals overlap values were reported relative to the surface area of a single carbon atom (36.3 Å^2^)[51]. Only glycans with relative overlaps (clash scores) of less than 1.0, corresponding to a single occluded carbon atom, were considered as satisfying the no-overlap criterion.

## Acknowledgments

The authors would like to thank Kausar N. Samli and Mari DeMarco for preparing samples for NMR analysis.

## Supporting Information

Figure S1 **Normalized experimental STD intensities for the TF disaccharide bound to JAA-F11 Fab.** (a). Theoretical STD intensities for the top four poses from docking (b-e); pose 1 shows the highest consistency with experimental STD data.
(TIF)

Figure S2 **STD-NMR intensity build up curves for a 200∶1 mixture of Galβ1-3GalNAc:JAA-F11 antibody, normalized to the intensity from the GalNAc N-Acetyl methyl group protons.** The experiment employed free disaccharide, and only the binding of the TF-α disaccharide was observed.
(DOC)

Table S1 **Docking results summary for the four pose clusters identified by docking the TF-disaccharide (Galβ1-3GalNAcα-OMe) to the FAB fragment of JAA-F11.** The lowest energy conformer from each cluster was used as a representative pose (Poses 1–4) in the CCG analysis.
(DOC)

Table S2 **Experimental values for the glycan array screening of JAA-F11.** Shown are the glycans containing the minimal binding determinant, Galβ1-3GalNAcα, and sequences that characterize the specificity of the mAb. ^a^Binding is considered to be present if the mean relative fluorescence signal is above at least 5% of the maximum signal in the sample at 200 ug/mL. ^b^Spacers are identified as follows: 0,–(CH_2_)_2_NH_2_; 8, –(CH_2_)_3_NH_2_; 14, threonine; 16, -p-nitrophenyl. ^c^Average value for redundant glycans on the v4.0 Glycan Array. ^d^Undefined anomeric configuration at reducing terminus.
(DOC)

Table S3 **Van der Waals overlaps from a CCG analysis of the CFG array glycans.** Greyed values indicate clash scores inconsistent with observed glycan array binding. Only Pose 1 is fully compatible with the experimental specificity data. Poses that lead to incompatibilities with the experimental data denoted with an asterisk (*). ^a^Binding or non-binding classification is based on a mean relative fluorescence signal greater than (binder) or less than (non-binder) 5% of the maximum signal for each concentration in array version 4.0. ^b^Sp0: –(CH2)2NH2; Sp8: –(CH2)3NH2. ^c^A methyl aglycon was employed in the grafting process to probe the effect of a β-linked spacer.
(DOC)

Results S1 **Corrections or Clarifications Associated with CFG v4.0 Glycan Array Annotations.** 1) When an anomeric center is not specified at the reducing end, the anomeric configuration is either not known or is present as a mixture. 2) The sequence for ligand **158** (CFG v4.0 ID) is Galβ1-3(Galβ1-4GlcNAcβ1-6) GalNAc-**Sp14**. The anomeric center is undetermined, or is a mixture, the spacer is number 14. 3) The sequence for ligand **159** is Galβ1-3(Galβ1-4GlcNAcβ1-6)GalNAc**α**-**Sp8**. The anomeric center is α, the spacer is 8. 4) Ligands **157** and **159** are identical, Galβ1-3(Galβ1-4GlcNAcβ1-6)GalNAcα-Sp8. 5) Ligands **125** and **182** are identical, Galβ1-3(GlcNAcβ1-6)GalNAcα-Sp8.
(DOC)
