# Supplementary figures and images for: Computational Screening of the Human TF-Glycome Provides a Structural Definition for the Specificity of Anti-Tumor Antibody JAA-F11
Source: PLoS One. 2013 Jan 24;8(1):e54874. doi: 10.1371/journal.pone.0054874 (PMC3554700; doi:10.1371/journal.pone.0054874)

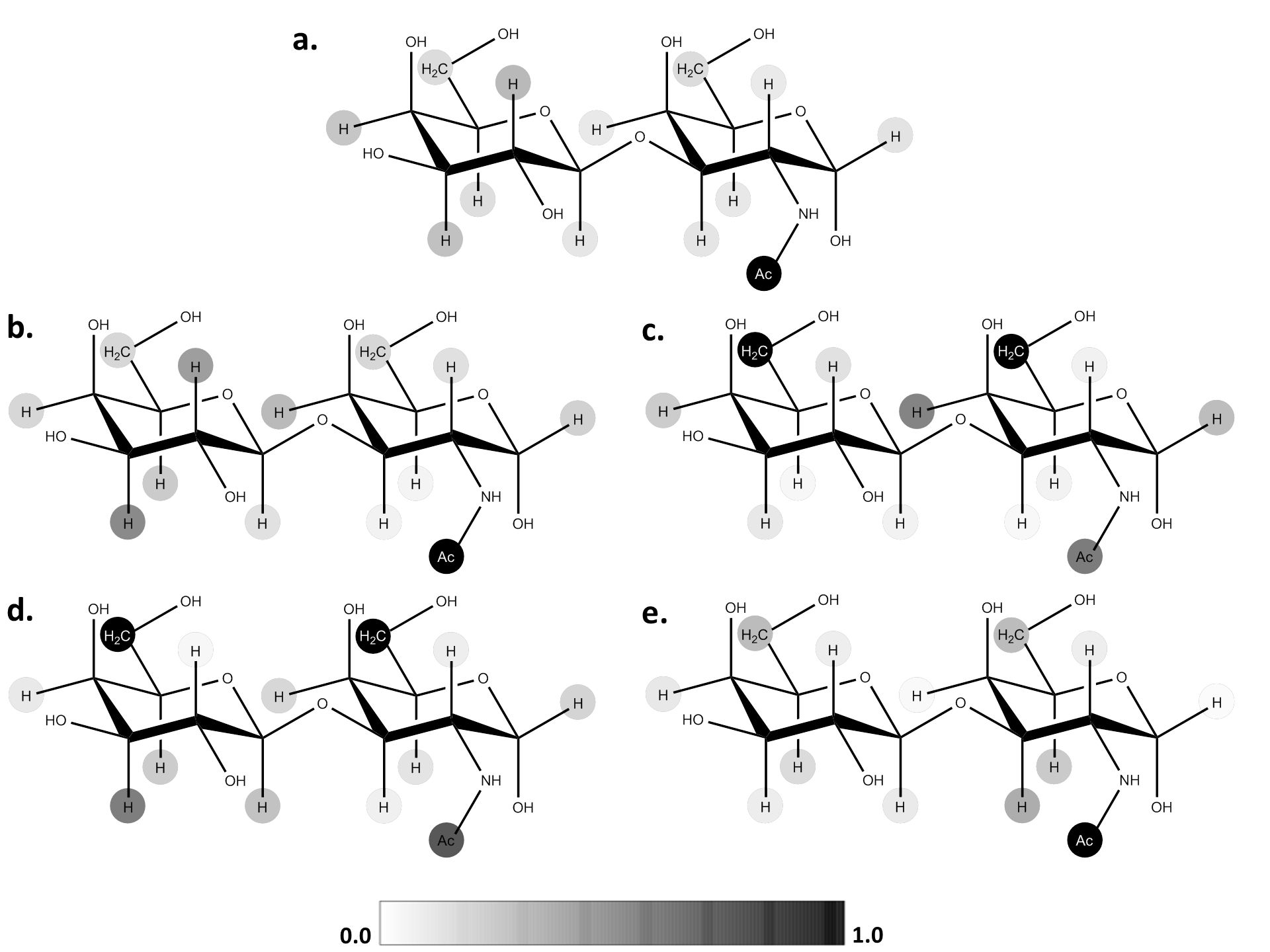

Supplement: Figure S1 — Normalized experimental STD intensities for the TF disaccharide bound to JAA-F11 Fab. (a). Theoretical STD intensities for the top four poses from docking (b-e); pose 1 shows the highest consistency with experimental STD data. (TIF) [file pone.0054874.s001.tif]

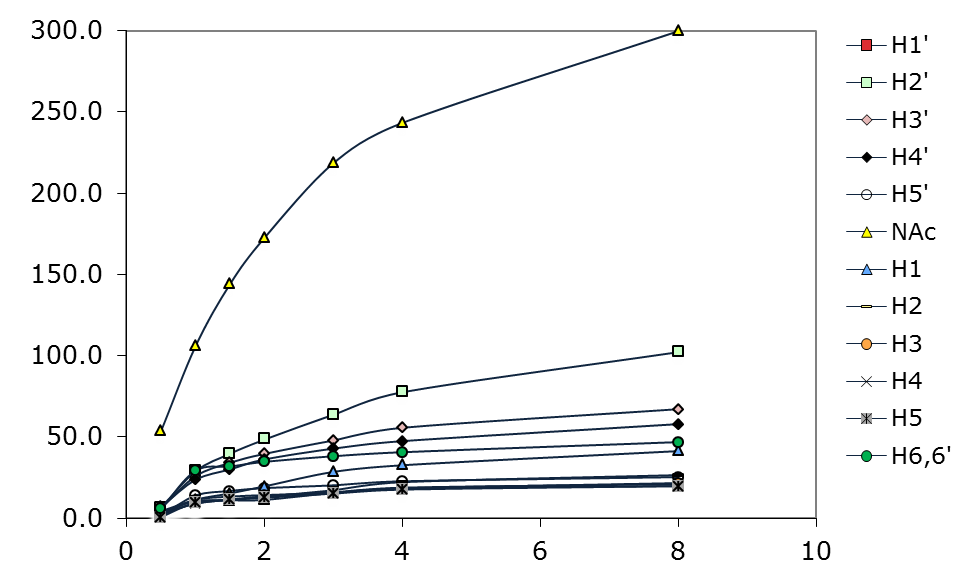

Supplement: Figure S2 — STD-NMR intensity build up curves for a 200∶1 mixture of Galβ1-3GalNAc:JAA-F11 antibody, normalized to the intensity from the GalNAc N-Acetyl methyl group protons. The experiment employed free disaccharide, and only the binding of the TF-α disaccharide was observed. (DOC) [file pone.0054874.s002.doc]
