# Supplementary material for: Computational Screening of the Human TF-Glycome Provides a Structural Definition for the Specificity of Anti-Tumor Antibody JAA-F11
Source: PLoS One. 2013 Jan 24;8(1):e54874. doi: 10.1371/journal.pone.0054874 (PMC3554700; doi:10.1371/journal.pone.0054874)
Supplement: Table S1 — Docking results summary for the four pose clusters identified by docking the TF-disaccharide (Galβ1-3GalNAcα-OMe) to the FAB fragment of JAA-F11. The lowest energy conformer from each cluster was used as a representative pose (Poses 1–4) in the CCG analysis. (DOC) [file pone.0054874.s003.doc]

| Cluster Rank^a^ | 1 | 2 | 3 | 4 |
| --- | --- | --- | --- | --- |
| Population (%) | 64 | 10 | 6 | 12 |
| Average docked energy^b^ | -8.8 | -8.0 | -7.3 | -6.7 |

^a^Based on a 2.0 Å cutoff. ^b^kcal mol^-1^.
