# Supplementary material for: Computational Screening of the Human TF-Glycome Provides a Structural Definition for the Specificity of Anti-Tumor Antibody JAA-F11
Source: PLoS One. 2013 Jan 24;8(1):e54874. doi: 10.1371/journal.pone.0054874 (PMC3554700; doi:10.1371/journal.pone.0054874)
Supplement: Table S2 — Experimental values for the glycan array screening of JAA-F11. Shown are the glycans containing the minimal binding determinant, Galβ1-3GalNAcα, and sequences that characterize the specificity of the mAb. aBinding is considered to be present if the mean relative fluorescence signal is above at least 5% of the maximum signal in the sample at 200 ug/mL. bSpacers are identified as follows: 0,–(CH2)2NH2; 8, –(CH2)3NH2; 14, threonine; 16, -p-nitrophenyl. cAverage value for redundant glycans on the v4.0 Glycan Array. dUndefined anomeric configuration at reducing terminus. (DOC) [file pone.0054874.s004.doc]

|  | Glycan^a^ |  | Mean Relative Fluorescence units (RFUs) | | |
| --- | --- | --- | --- | --- | --- |
|  |  | Sp^b^ | 0.1 µg/mL | 5  µg/mL | 200  µg/mL |
| 1 | **Galβ1-3GalNAcα** | 8 | 16223 | 39364 | 58481 |
|  |  | 14 | 8 | 30 | 14 |
|  |  | 16 | 15 | 28 | 15565 |
| 2 | Neu5Acβ2-6(**Galβ1-3**)**GalNAcα** | 8 | 12388 | 33481 | 52238 |
| 3 | Neu5Acα2-6(**Galβ1-3**)**GalNAcα** | 8 | 13105 | 32998 | 45410 |
|  |  | 14 | 7 | 11 | 6 |
| 4 | Galβ1-4GlcNAcβ1-6(**Galβ1-3**)**GalNAcα** | 8^c^ | 2023 | 18060 | 62789 |
|  |  | 14^d^ | -2 | 11 | 1 |
| 5 | GlcNAcβ1-6(**Galβ1-3**)**GalNAcα** | 8 | 2357 | 16637 | 61857 |
|  |  | 14^d^ | -1 | 12 | 8 |
| 6 | Galα1-3GalNAcα | 8 | 17 | 256 | 45611 |
|  |  | 16 | 2 | 8 | 8 |
| 7 | Galβ | 8 | 12 | 2 | 45 |
| 10 | Fucα1-2**Galβ1-3GalNAcα** | 8 | -5 | 17 | 94 |
|  |  | 14 | 4 | 31 | 24 |
| 11 | GlcNAcβ1-3**Galβ1-3GalNAcα** | 8 | 17 | 29 | 53 |
| 12 | Neu5Acα2-3Galβ1-4(Fucα1-3)GlcNAcβ1-6(**Galβ1-3**)**GalNAcα** | 14 | 31 | 48 | 42 |
| 13 | Neu5Acα2-3Galβ1-4GlcNAcβ1-6(**Galβ1-3**)**GalNAcα** | 14 | -1 | 21 | 21 |
| 14 | Fucα1-2**Galβ1-3GalNAcα**1-3(Fucα1-2)Galβ1-4Glcβ | 0 | 5 | 23 | 20 |
| 15 | GlcNAcα1-4**Galβ1-3GalNAc** | 14^d^ | 10 | 48 | 18 |
| 16 | GlcNAcβ1-2**Galβ1-3GalNAcα** | 8 | 5 | 23 | 17 |
| 17 | Neu5Acα2-3Galβ1-4(Fucα1-3)GlcNAcβ1-6(Neu5Acα2-3**Galβ1-3**)**GalNAc** | 14^d^ | 38 | 35 | 14 |
| 18 | GalNAcα1-3(Fucα1-2)**Galβ1-3GalNAcα** 1-3(Fucα1-2)Galβ1-4GlcNAcβ | 0 | 1 | 8 | 14 |
| 19 | **Galβ1-3GalNAcα**1-3(Fucα1-2) Galβ1-4GlcNAc | 0^d^ | 8 | 10 | 14 |
| 20 | **Galβ1-3GalNAcα**1-3(Fucα1-2)Galβ1-4Glc | 0^d^ | 13 | 19 | 13 |
| 21 | (3S)**Galβ1-3GalNAcα** | 8 | -2 | 6 | 12 |
| 22 | Fucα1-2**Galβ1-3GalNAcα**1-3(Fucα1-2)Galβ1-4GlcNAcβ | 0 | 1 | 17 | 10 |
| 23 | Neu5Acα2-6(Neu5Acα2-3**Galβ1-3**)**GalNAcα** | 8 | 2 | 23 | 8 |
|  |  | 14 | 19 | 22 | 12 |
| 24 | 6S(Neu5Acα2-3**Galβ1-3)GalNAcα** | 8 | 8 | 19 | 7 |
| 25 | Neu5Acα2-3**Galβ1-3GalNAcα** | 8 | 5 | 14 | 5 |
|  |  | 14 | 4 | 7 | 6 |
| 26 | Neu5Acα2-3Galβ1-4GlcNAcβ1-6(Neu5Acα2-3**Galβ1-3**)**GalNAcα** | 14 | 19 | 11 | 2 |
| 27 | KDNα2-3**Galβ1-3GalNAcα** | 14 | -8 | 6 | 0 |
