# Supplementary material for: Computational Screening of the Human TF-Glycome Provides a Structural Definition for the Specificity of Anti-Tumor Antibody JAA-F11
Source: PLoS One. 2013 Jan 24;8(1):e54874. doi: 10.1371/journal.pone.0054874 (PMC3554700; doi:10.1371/journal.pone.0054874)
Supplement: Table S3 — Van der Waals overlaps from a CCG analysis of the CFG array glycans. Greyed values indicate clash scores inconsistent with observed glycan array binding. Only Pose 1 is fully compatible with the experimental specificity data. Poses that lead to incompatibilities with the experimental data denoted with an asterisk (*). aBinding or non-binding classification is based on a mean relative fluorescence signal greater than (binder) or less than (non-binder) 5% of the maximum signal for each concentration in array version 4.0. bSp0: –(CH2)2NH2; Sp8: –(CH2)3NH2. cA methyl aglycon was employed in the grafting process to probe the effect of a β-linked spacer. (DOC) [file pone.0054874.s005.doc]

| **ID** | **CFG ID v4.0** | **Binders^a^** | **Sp^b^** | **Pose 1** | **Pose 2** | **Pose 3** | **Pose 4** |
| --- | --- | --- | --- | --- | --- | --- | --- |
| 1 | 131 | **Galβ1-3GalNAcα** | 8 | 0 | 0 | 0 | 0 |
| 2 | 129 | Neu5Acβ2-6(**Galβ1-3**)**GalNAcα** | 8 | 0 | 0.7 | 5.9* | 0 |
| 3 | 127 | Neu5Acα2-6(**Galβ1-3**)**GalNAcα** | 8 | 0.1 | 1.1* | 10* | 0 |
| 4 | 157,159 | Galβ1-4GlcNAcβ1-6(**Galβ1-3**)**GalNAcα** | 8 | 0 | 0 | 13.1* | 0 |
| 5 | 125,182 | GlcNAcβ1-6(**Galβ1-3**)**GalNAcα** | 8 | 0 | 0 | 5.1* | 0 |
|  |  | **Non-Binders^a^** |  |  |  |  |  |
| 10 | 58 | Fucα1-2**Galβ1-3GalNAcα** | 8 | 7.6 | 1.2 | 0* | 7 |
| 11 | 85 | GlcNAcβ1-3**Galβ1-3GalNAcα** | 8 | 12.4 | 12.3 | 0* | 8.7 |
| 16 | 166 | GlcNAcβ1-2**Galβ1-3GalNAcα** | 8 | 11.6 | 3.4 | 0* | 9.8 |
| 21 | 27 | (3S)**Galβ1-3GalNAcα** | 8 | 2.8 | 0* | 0* | 2.3 |
| 23 | 233 | Neu5Acα2-6(Neu5Acα2-3**Galβ1-3**)**GalNAcα** | 8 | 17.6 | 17.7 | 9.4 | 12.4 |
| 25 | 232 | 6S(Neu5Acα2-3**Galβ1-3**)**GalNAcα** | 8 | 17.3 | 16.7 | 0.7* | 14.2 |
| 26 | 214 | Neu5Acα2-3**Galβ1-3GalNAcα** | 8 | 17.4 | 16.8 | 0* | 12.4 |
| 31 | 91 | **Gal**NAc**β1-3GalNAcα** | 8 | 2.5 | 0.3* | 0* | 2 |
| 32 | 133 | **Galβ1-3GalNAc**β-Sp8^c^ | 8 | 2.2 | 1.3 | 3.1 | 0* |
| 33 | 134 | **Galβ1-3GalNAc**β1-4Galβ1-4Glcβ | 0 | 15.6 | 17 | 21.1 | 0* |
