# Supplementary material for: Computational Screening of the Human TF-Glycome Provides a Structural Definition for the Specificity of Anti-Tumor Antibody JAA-F11
Source: PLoS One. 2013 Jan 24;8(1):e54874. doi: 10.1371/journal.pone.0054874 (PMC3554700; doi:10.1371/journal.pone.0054874)
Supplement: Results S1 — Corrections or Clarifications Associated with CFG v4.0 Glycan Array Annotations. 1) When an anomeric center is not specified at the reducing end, the anomeric configuration is either not known or is present as a mixture. 2) The sequence for ligand 158 (CFG v4.0 ID) is Galβ1-3(Galβ1-4GlcNAcβ1-6) GalNAc-Sp14. The anomeric center is undetermined, or is a mixture, the spacer is number 14. 3) The sequence for ligand 159 is Galβ1-3(Galβ1-4GlcNAcβ1-6)GalNAcα-Sp8. The anomeric center is α, the spacer is 8. 4) Ligands 157 and 159 are identical, Galβ1-3(Galβ1-4GlcNAcβ1-6)GalNAcα-Sp8. 5) Ligands 125 and 182 are identical, Galβ1-3(GlcNAcβ1-6)GalNAcα-Sp8. (DOC) [file pone.0054874.s006.doc]

**SUPPLEMENTARY RESULTS**

*Corrections or Clarifications Associated with CFG v4.0 Glycan Array Annotations*

1) When an anomeric center is not specified at the reducing end, the anomeric configuration is either not known or is present as a mixture.

2) The sequence for ligand **158** (CFG v4.0 ID) is Galβ1-3(Galβ1-4GlcNAcβ1-6) GalNAc-**Sp14**. The anomeric center is undetermined, or is a mixture, the spacer is number 14.

3) The sequence for ligand **159** is Galβ1-3(Galβ1-4GlcNAcβ1-6)GalNAc**α**-**Sp8**. The anomeric center is α, the spacer is 8.

4) Ligands **157** and **159** are identical, Galβ1-3(Galβ1-4GlcNAcβ1-6)GalNAcα-Sp8.

5) Ligands **125** and **182** are identical, Galβ1-3(GlcNAcβ1-6)GalNAcα-Sp8.
